# Pelvis of *Gargoyleosaurus* (Dinosauria: Ankylosauria) and the Origin and Evolution of the Ankylosaur Pelvis

**DOI:** 10.1371/journal.pone.0079887

**Published:** 2013-11-14

**Authors:** Kenneth Carpenter, Tony DiCroce, Billy Kinneer, Robert Simon

**Affiliations:** 1 Prehistoric Museum, Utah State University – Eastern, Price, Utah, United States of America; 2 Geology Section, University of Colorado Museum, Boulder, Colorado, United States of America; 3 Denver Museum of Nature and Science, Denver, Colorado, United States of America; 4 Dinosaur Safaris Inc., Ashland, Virginia, United States of America; Raymond M. Alf Museum of Paleontology, United States of America

## Abstract

Discovery of a pelvis attributed to the Late Jurassic armor-plated dinosaur *Gargoyleosaurus* sheds new light on the origin of the peculiar non-vertical, broad, flaring pelvis of ankylosaurs. It further substantiates separation of the two ankylosaurs from the Morrison Formation of the western United States, *Gargoyleosaurus* and *Mymoorapelta*. Although horizontally oriented and lacking the medial curve of the preacetabular process seen in *Mymoorapelta*, the new ilium shows little of the lateral flaring seen in the pelvis of Cretaceous ankylosaurs. Comparison with the basal thyreophoran *Scelidosaurus* demonstrates that the ilium in ankylosaurs did not develop entirely by lateral rotation as is commonly believed. Rather, the preacetabular process rotated medially and ventrally and the postacetabular process rotated in opposition, i.e., lateral and ventrally. Thus, the dorsal surfaces of the preacetabular and postacetabular processes are not homologous. In contrast, a series of juvenile *Stegosaurus* ilia show that the postacetabular process rotated dorsally ontogenetically. Thus, the pelvis of the two major types of Thyreophora most likely developed independently. Examination of other ornithischians show that a non-vertical ilium had developed independently in several different lineages, including ceratopsids, pachycephalosaurs, and iguanodonts. Therefore, a separate origin for the non-vertical ilium in stegosaurs and ankylosaurs does have precedent.

## Introduction

Armor-plated ankylosaurs are a minor component of the Late Cretaceous landscape, except in the arid paleoenvironments of central Asia. They are characterized by a broad, flaring pelvis that can be wider than long, formed by nearly horizontal ilia. The origin of this odd pelvis has been variously attributed to the protection of internal organs [1] or support of heavy dermal armor [2]. These hypotheses are examined in light of the discovery of the pelvis attributed to the primitive ankylosaur *Gargoyleosaurus parkpinorum* from the Upper Jurassic Morrison Formation in the Big Horn Basin, Wyoming. Taken in context with the older *Scelidosaurus* from the Lower Jurassic of England, and the younger *Euoplocephalus* from the Cretaceous of North America, the pelvis of *Gargoyleosaurus* is intermediate in its morphology.

Traditionally, the non-vertical ilium of ankylosaurs was interpreted as forming from lateral folding of the ilium so that the lateral surface faced ventrally [1], [2], although no evidence was presented. This “self-evident” interpretation is widely accepted today [3], [4]. Our study below of ankylosaur pelvic origins and evolution casts doubt on this interpretation, and has major ramifications on the phylogenetics of the Thyreophora.

## Methods

Specimens were studied and photographed in their respective institutions. There were no field studies; therefore, no permits were required.

## Materials

Institutional Abbreviations: AMNH – American Museum of Natural History, New York, USA); ANSP – Academy of Natural Sciences (Philadelphia, PA); BHI – Black Hills Institute of Geological Research (Hill City, SD). BMAG – Bristol Museum and Art Gallery (Bristol, UK); BMNH – Natural History Museum of London (London, UK); CMNH – Carnegie Museum of Natural History (Pittsburgh, PA); DINO – Dinosaur National Monument (Jensen, UT); DMNH – Denver Museum of Science and Nature (Denver, Colorado, USA); HMNH – Hayashibara Museum of Natural History (Okayama, Japan); IGM - Institute of Geology, Mongolian Academy of Sciences (Ulaan Baatar, Mongolia). MNA – Museum of Northern Arizona (Flagstaff, AZ); MWC – Museum of Western Colorado (Grand Junction, Colorado, USA); NMC – National Museums of Canada (Ottawa, ON); SAM, South African Museum, Cape Town, South Africa; SGDS – Saint George Dinosaur Center (Saint George, Utah, USA).

Specimens And Their Localities: *Gargoyleosaurus parkpinorum*: partial pelvis (DMNS 58831) comprised of a complete synsacrum, with damaged neural spines, articulated with a complete right ilium, from the Morrison Formation, Simon Quarry, Big Horn County, Wyoming, USA. Nearly complete right pubis with associated partial skeleton (DMNH 27726) from the Morrison Formation, Bone Cabin Quarry West, Albany County, Wyoming USA. Pelvis parts of other specimens examined include: *Camptosaurus amphanoecetes* pelvis from a nearly complete skeleton (CM 11337), Morrison Formation, Dinosaur National Monument, Utah, USA. *Chasmosaurus belli* pelvis (ANSP 15764, cast of NMC 2245), Dinosaur Park Formation, Dinosaur Provincial Park, Alberta, Canada. *Dryosaurus altus* pelvis (CM 3392), Morrison Formation, Dinosaur National Monument, Utah, USA. *Edmontonia rugosidens* ischia and pubis associated with holotype partial skeleton (USNM 11868), Two Medicine Formation, Glacier County, Montana, USA; nearly complete pelvis in dorsal view (AMNH FR5381), Dinosaur Park Formation, Dinosaur Provincial Park, Alberta, Canada. *Edmontonia longiceps* pelvis in ventral view (NMC 8531), Horseshoe Canyon Formation, Alberta, Canada. *Edmontonia schlessmani* (BHI 127327), Lance Formation, Niobrara County, Wyoming, USA. *Edmontonosaurus annectens* pelvis (BHI 126,414), Hell Creek Formation, Ziebach County, South Dakota, USA. *Euoplocephalus tutus* complete pelvis of a partial skeleton (AMNH FR5337), Dinosaur Park Formation, Dinosaur Provincial Park, Alberta, Canada. *Homalocephale calathoceros* ilium of a partial skeleton (IGM 100/51), Nemegt Formation, Nemegt Basin, Mongolia. *Lesothosaurus diagnosticus* right ilium, pubis and ischium (BMNH RU 17), Elliot Formation, Likhoele, Lesotho. *Mymoorapelta maysii* complete left ilium (MWC 1815) and left ischium (MWC 4027), Morrison Formation, Mygatt-Moore Quarry, Mesa County, Colorado, USA; natural mold of the sacral vertebrae, proximal end of the ischium and partial pubis (MWC 2610), Morrison Formation, Hups Quarry, Mesa County, Colorado, USA. *Planicoxa depressus* ilium (USNM 4759), Lakota Sandstone, Calico Canyon, South Dakota, USA. *Saichania chulsanensis* pelvis of nearly complete skeleton (IGM 100/1305), Barun Goyot Formation, Khulsan, Nemegt Basin, Mongolia. *Scelidosaurus harrisoni* complete pelvis of a nearly complete skeleton of (BMNH R1111), Charmouth Mudstone, Charmouth, England. Right ilium of a partial juvenile skeleton (BMNH R6704) from the Black Ven Marls, Charmouth, England; left pelvis of a complete skeleton (SGDS 1311 cast of BMAG uncataloged specimen B), Black Ven Marls, Charmouth, England. *Scutellosaurus lawleri* pelvic region of holotype (MNA Pl. 175), Kayenta Formation, Ward Terrace, Arizona, USA. *Stegosaurus* cf. *S. stenops*: pelvis of a skeleton (DMNH 1438), Morrison Formation, Garden Park, Colorado, USA; nearly complete right ilium of very small juvenile (DMNH 33359), Morrison Formation, Bone Cabin Quarry West, Albany County, Wyoming, USA; complete right ilium of a partial juvenile skeleton (DMNH 33360 cast of DINO 2438), Morrison Formation, Dinosaur National Monument, Utah, USA; *Stormbergia dangershoeki* right pubis and ischium (SAM-PK-K1105), Upper Elliot Formation, Dangershoek Farm, South Africa. *Thescelosaurus neglectus* pelvis associated with partial skeleton (AMNH FR117), Lance Formation, Wyoming, or Hell Creek Formation, South Dakota. Unnamed ankylosaurid (HMNS97-21-1), Djadokhta Formation, Abdrant Nuru, Mongolia. Much of the terminology for the ilium is expanded from [5].

## Results and Discussion

### Description

**Figure 1:**
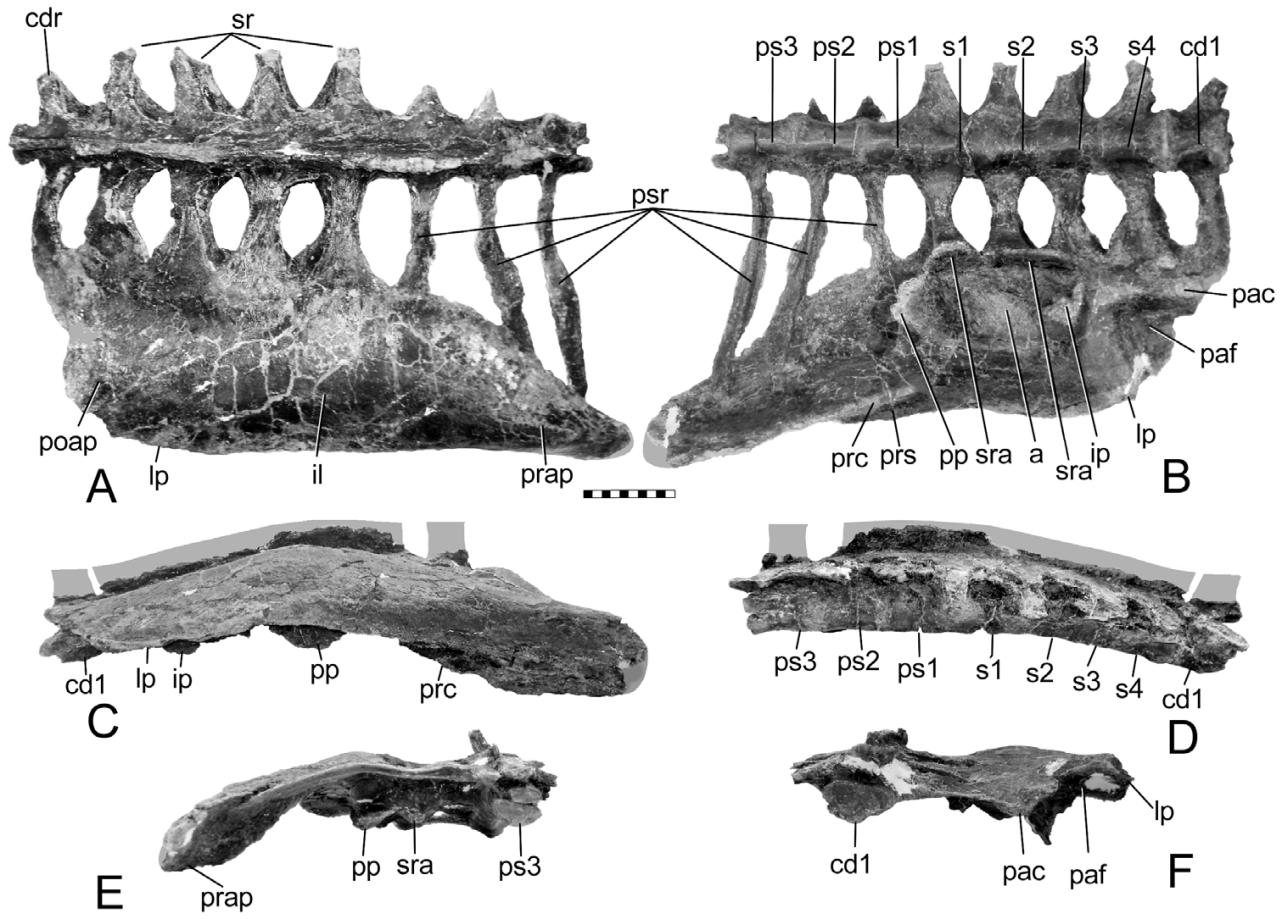
New pelvis of *Gargoyleosaurus parkpinorum* (DMNH 58831). Views in: A, dorsal; B, ventral; C, right lateral; D, left lateral; E, anterior; and F, posterior. Scales in cm. Abbreviations: a – acetabulum; cd1 – caudal vertebra 1; cdr – caudal rib; ip – ischial peduncle; lp – lateral process; pac – postacetabular crest; paf – postacetabular fossa; poap – postacetabular process; pp – pubic penducle; prap - preacetabular process; prc – preacetabular crest; prs – preacetabular shelf; ps1-3 – presacral vertebrae 1-3; psr – presacral vertebrae ribs; s1-4 – sacral vertebrae 1-4; sr – sacral rib(s); sra – sacral rib backing of acetabulum.

**Figure 2:**
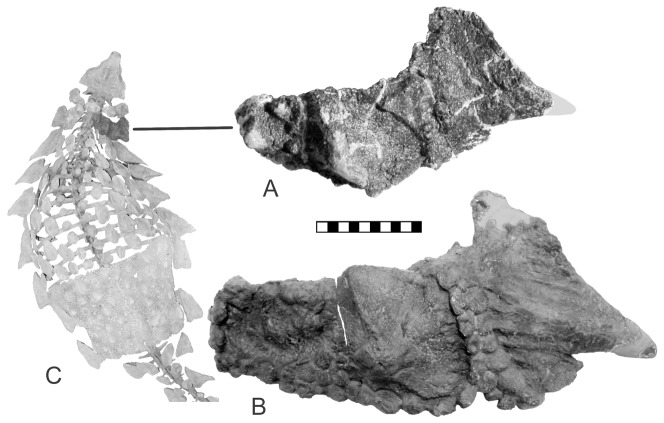
Cervical half-rings of *Gargoyleosaurus*. A, holotype specimen (DMNH 27726) shown on a mounted skeleton of the holotype; B, referred specimen (DMNH 58831). Note that both share having three osteoderms arranged in an arc. The only significant difference are the smaller osteoderms that have attached themselves along the posterior side of larger specimen; this is probably ontogenetic. Scale in cm.

The new pelvis (Fig. 1) is referable to *Gargoyleosaurus* [6], [7] because the associated right cervical quarter-ring osteoderms matches the same quarter-ring of the holotype, DMNH 27726 (Fig. 2). The utility of osteoderms in ankylosaur taxonomy has been discussed previously [8]–[11]. Both quarter-rings are composed of two coossified keeled osteoderms that are coossified to a posterolaterally projecting, dorso-ventrally compressed triangular plate laterally, and partially to a thin bone band ventrally; a small triangular gap partially separates the lateral triangular plate from the bone band. The sutural zones are marked by small ossicles that are coossified to the larger osteoderms. These ossicles are more extensively developed along the posterior side of the referred quarter-ring, which because of its larger size as compared to the holotype quarter-ring, is probably ontogenetic (i.e., increased ossification with age). The other ankylosaur from the Morrison Formation, *Mymoorapelta* [12] shares with *Gargoyleosaurus* the triangular, dorsoventrally compressed lateral plates. However, although *Mymoorapelta* apparently has rows of cervical osteoderms, these are not coossified into a quarter-ring or half-ring despite having been reconstructed as such on the skeletal mount (Museum of Western Colorado; the skull on the mount is modeled on *Gargoyleosaurus* as well).

The synsacrum of DMNS 58831is composed of four sacral vertebrae (defined below), one caudal vertebra and three presacral vertebrae (Fig. 1B). The term “presacral” is used here as originally defined by Osborn ([13]:193): “It is best to enumerate the dorsals also from the sacrum forwards, namely as presacrals 1, 2, 3, etc.” This numerical system was introduced because the number of dorsal vertebrae was unknown for the specimen (*Diplodocus*) that he was describing. Osborn pointedly did not include the cervicals. Nevertheless, in recent years the term “presacral vertebrae” has been misused, with presacral vertebra 1 being equal to either the first cervical or first dorsal vertebrae. As so used, the term is redundant with cervical 1 or dorsal 1. The presacral centra in *Gargoyleosaurus* are spool-shaped and slightly compressed laterally so as to be taller than wide. The centra of the first presacral, the four sacrals and first caudal of *Gargoyleosaurus* are ventrolaterally constricted forming a midline keel, whereas the centra of presacral 2 and 3 are rounded and the keel absent. The caudal centrum is wider than long, whereas the rest of the centra in the synsacrum are longer than wide. The ribs of the three presacrals are coossified to their respective neural arches. In addition, the rib of presacral 1 is coossified to the medial edge of the preacetabular process, and the ribs of presacrals 2 and 3 underlap and are fused to the ventral side of the preacetabular process as in other ankylosaurs, including *Mymoorapelta*.

**Figure 3:**
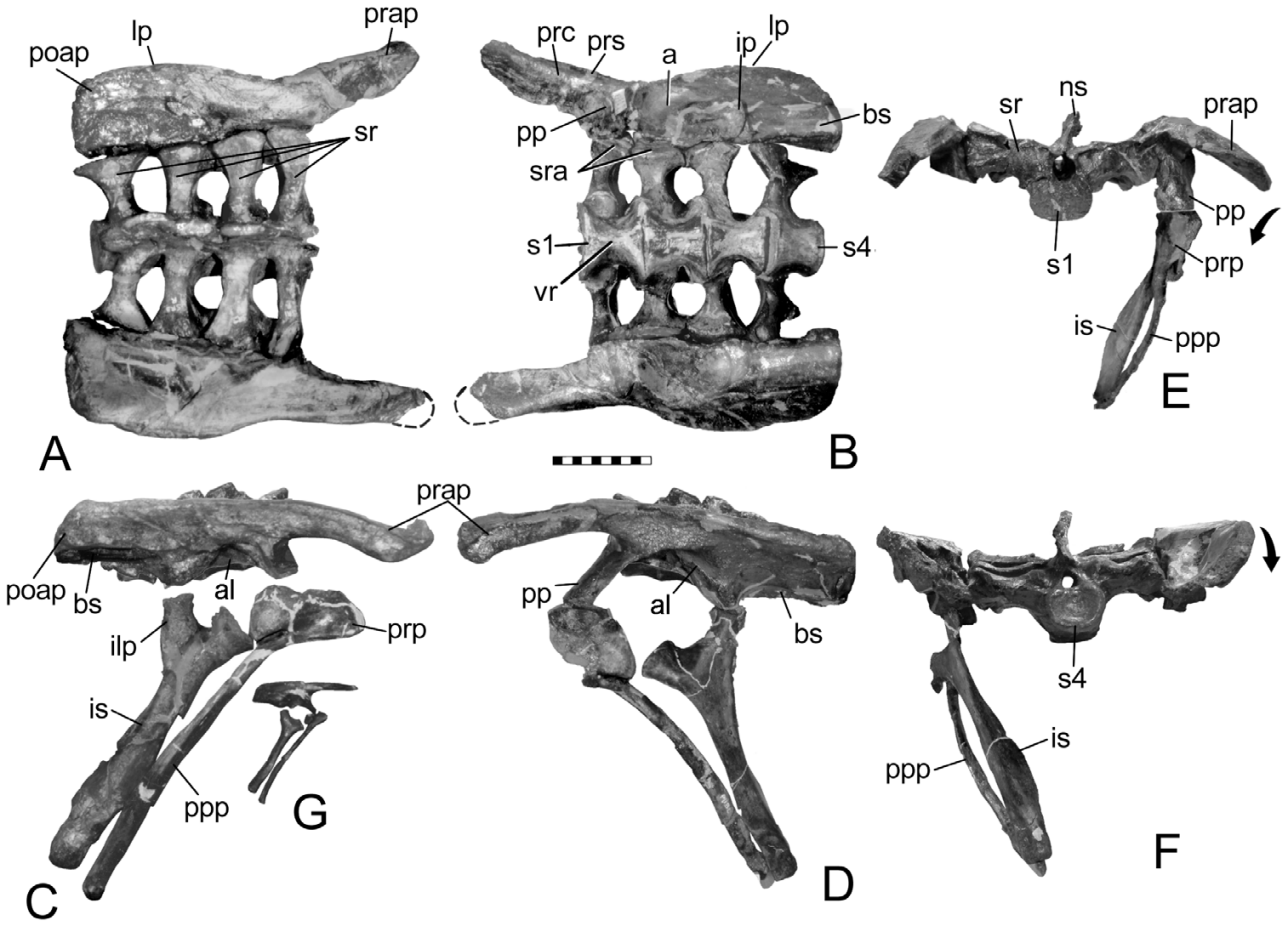
Pelvis of *Scelidosaurus harrisoni* (BMNH R1111). Views in: A, dorsal; B, ventral; C, right lateral; D, left lateral; E, anterior; and F, posterior. G, juvenile pelvis (BMNH 6704). In E, the prepacetabular process can be seen rotated (arrow) so that the medial surface faces ventromedially, and in F, the postacetabular process has rotated in opposition (arrow); See also Fig. 10. Scale in cm. Abbreviations: a – acetabulum; al – acetabular lamina; bs – brevis shelf; ilp – ilial peduncle; ip – ischial peduncle; is – ischium; lp – lateral process; ns – neural spine; poap – postacetabular process; pp – pubic penducle; ppp – postpubic process; prap - preacetabular process; prc – preacetabular crest; prp – prepubic process; prs – preacetabular shelf; s1-4 – sacral vertebrae 1–4; sr – sacral rib(s); sra – sacral rib backing of acetabulum; vr – ventral ridge.

The sacral vertebrae are identified based on the criteria set forth by Owen [14], [15] based primarily on their ribs, which are short, deep, extend in part from or between the centra, and contact one another distally to brace the ilium on its medial side (Fig. 1B). By this definition, not every rib that contacts the ilium is a sacral rib. As with *Scelidosaurus*, sacral ribs 1 and 2 contribute to the ventral margin of the acetabulum (Fig. 3), and additionally have some additional contribution by sacral rib 3 (Fig. 1B). Sacral rib 4 only articulates medially with the postacetabular process. The sacral ribs slightly decrease in length (∼10%) posteriorly from the first to the fourth, so that the sacral yoke is angled eight degrees anterolaterally. There is no closure of the fenestrae between the sacral ribs as in some stegosaurs (e.g., *Stegosaurus*, but not *Huayangosaurus*). Although the neural spines are damaged, what remains of their bases shows that the spine of presacral 3 was separate, rather than coossified with the neural spines of the other two presacrals and sacrals into a vertical plate.

**Figure 4:**
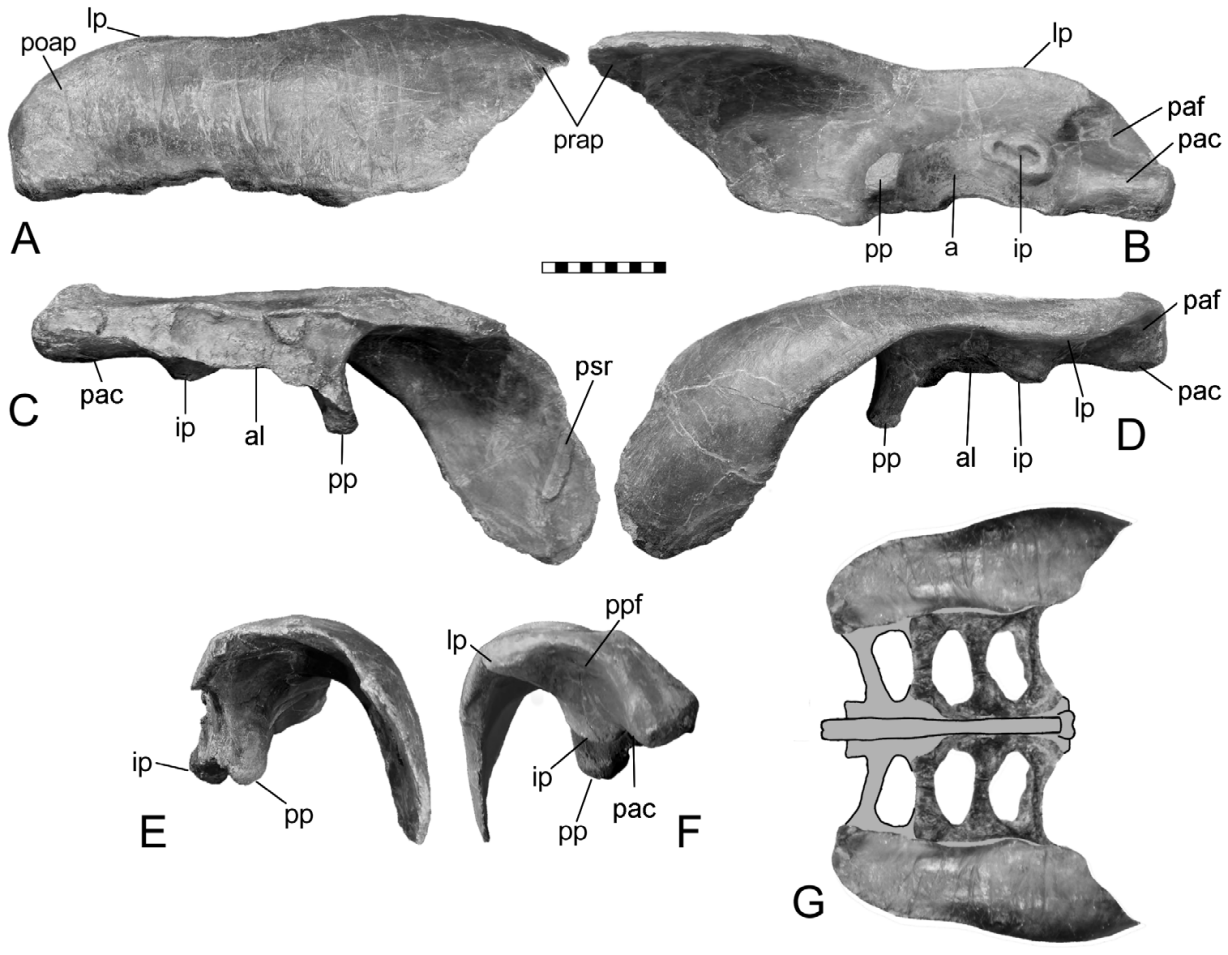
Holotype left ilium of *Mymoorapelta maysii* (MWC 1815). Views in: A, dorsal; B, ventral; C, medial; D, left lateral; E, anterior; and F, posterior. G, reconstruction of pelvis in dorsal view using the partial sacrum of MWC 2610 (not to scale with A–F). Scale in cm. Abbreviations: a – acetabulum; al – acetabular lamina; ip – ischial peduncle; lp – lateral process; pac – postacetabular crest; paf – postacetabular fossa; poap – postacetabular process; pp – pubic penducle; prap - preacetabular process; psr – preasacral vertebra rib.

The ilium is a large, subrectangular, subhorizontal plate, convex dorsally and concave ventrally. It is parallel sided, except for the tapering preacetabular process, which curves medioventrally (Fig. 1C) to overlay the posterior presacral ribs. The long axis of the ilium is parallel to the sacrals, as in *Mymoorapelta* (Fig. 4), except the anterior portion where the medial edge angles anterolaterally. The lateral process or supra-acetabular crest (“antitrochanter” of [1], often erroneously given today without the quotation marks implying homology with the antitrochanter in birds) is a slight projection on the posterolateral corner of the postacetabular process. It connects to the ischial peduncle by a low ridge as in stegosaurs and hadrosaurs.

**Figure 5:**
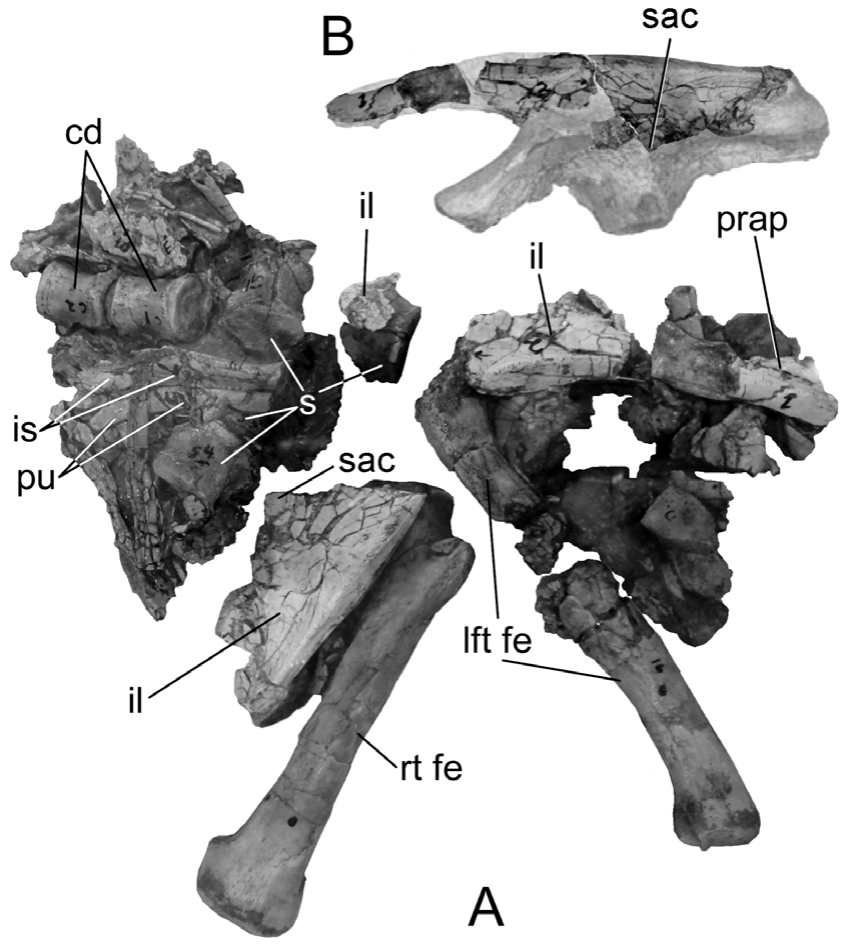
Holotype pelvic region of *Scutellosaurus lawleri* (MNA Pl. 175). A, right lateral view. B, ilium parts best fit on the ilium of *Stormbergia*. Scale in cm. Abbreviations: cd – caudal vertebrae; il – ilium fragments; is  =  ischium fragments; lft fe – left femur; pu – pubic fragments; prap - preacetabular process; rt fe – right femur; s – sacral vertebrae; sac – supra-acetebular crest.

In lateral view, the ilium is slightly bowed, with the highest point above the pubic peduncle. In contrast, the ilium of *Mymoorapelta* is flat in lateral view (Fig. 4C, D). Ventrally, the acetabulum is formed between the moderately long pubic peduncle and squat, swollen ischial peduncle. It is located about midway between the medial and lateral margins of the ilium. The axis of the acetabulum is angled 50° posteriorly from an axis through the middle of the acetabulum perpendicular to the sacral vertebrae. This angle causes the femur to swing anterolaterally so that the knee clears the expanded gut; the femur does not move in a parasagittal plane. The preacetabular process has a crest ventrally that extends forwards from near the pubic peduncle. Anteriorly, this crest becomes the ventral margin of the preacetabular process. Near the pubic peduncle, this preacetabular crest separates a narrow shelf (the preacetabular shelf) from the main body of the preacetabular process. This shelf may have been for the origin for part of the M. iliotibialis. The preacetabular shelf and associated crest are present in *Scelidosaurus*, although are not as well developed. They are absent in *Huayangosaurus* and most stegosaurs, except *Dacentrurus* (BMNH 46013) and an unnamed *Dacentrurus*-like pelvis from China ([16] fig. 5f).

The pubic peduncle is deformed, having been bent forwards. Nevertheless, it is less robust as compared to *Scelidosaurus* and *Mymoorapelta* (compare Figs. 1, 3, 4). The peduncle lacks the primitive cylindrical structure seen in *Stormbergia*, *Scelidosaurus* and *Mymoorapelta*. Like later ankylosaurs, it forms a wall along the anterior width of the acetabulum. The ischial peduncle is short and broadly swollen, unlike the more prominent condition seen in *Stormbergia* or *Lesothosaurus*. A broad, low ridge, or postacetabular crest, extends posteriorly from the ischial peduncle along the medial side of the postacetabular process to the distal end. No brevis shelf or fossa is present medially for the caudofemoralis brevis, unlike *Scelidosaurus* (Fig. 3B). Instead, the muscle probably originated from the side of the postacetabular crest and the adjacent portions of the posterior synsacral ribs. A triangular fossa is formed between the postacetabular crest and the ridge extending from the lateral process and ischial peduncle. The M. flexor tibialis externus probably originated there.

### Origin and Evolution of the Thyreophoran Pelvis

**Figure 6:**
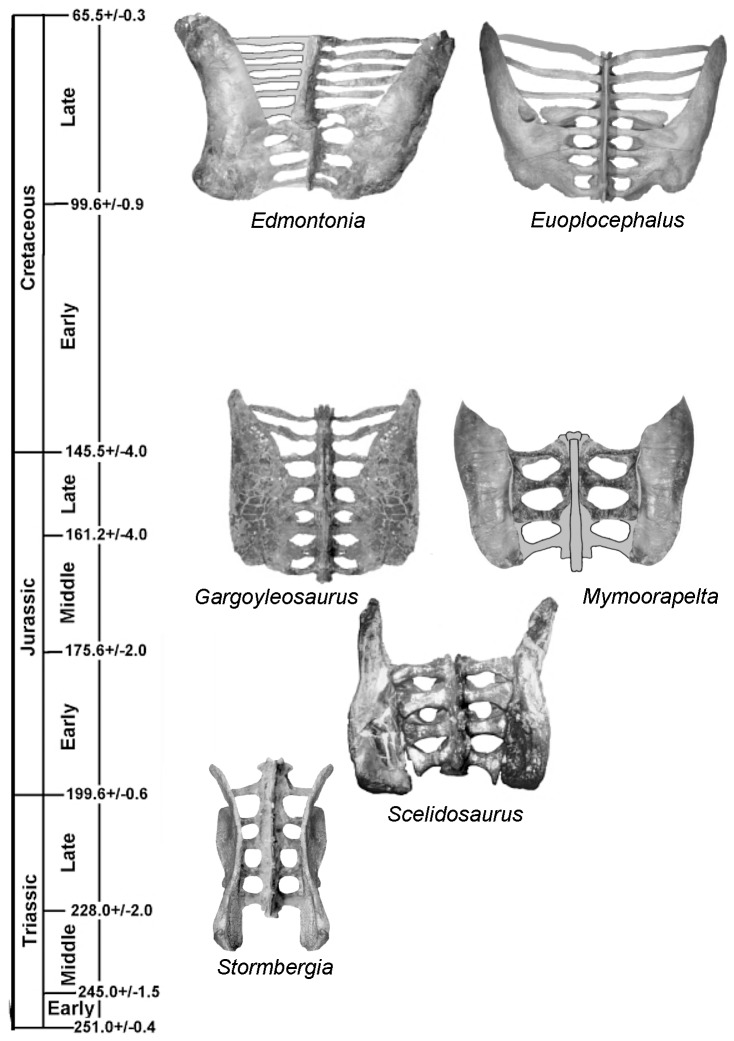
The origin and evolution of the ankylosaur pelvis can be approximated from this chronostratigraphic distribution of various pelves seen in dorsal view. In the basal ornithischian condition, represented by *Stormbergia* (reconstructed), the ilia are near vertical plates of bone. In the earliest ankylosauromorph, respresented by *Scelidosaurus*, the dorsal rim of the iliac blade and postacetabulum have rotated towards the lateral side so as to overhang the femur head, and an incipient synsacrum developed. In addition, the elongation of the sacral ribs is accompanied by the medial rotation of the preacetabular process. In the earliest ankylosaur, represented by *Gargoyleosaurus*, the ilium has assumed a nearly horizontal position and a synsacrum was developed including both caudals and posterior dorsals. In contrast, the preacetabulum of *Mymoorapelta* curved ventrally for reasons not clear; regardless, this specialization suggests that *Mymoorapelta* is not the close sister taxon to later ankylosaurs. Further evolution of the ankylosaur pelvis resulted in divergence of the ilia, seen in nodosaurids, represented by *Edmontonia*, but especially in ankylosaurids, represented by *Euoplocephalus*. In addition, there was further elongation of the sacral ribs. Not to scale. See also Fig. 10.

To set the pelvis of *Gargoyleosaurus* into context of ankylosaur pelvic evolution, the pelvis of several other taxa need to be redescribed and illustrated. The pelvis has been described in general terms [2], [3], [17], [18], as well as for specific taxa [19]-[23]. Stegosaurs and ankylosaurs form the clade Thyreophora [24], along with *Scutellosaurus* and *Scelidosaurus* [25]. The placement of *Lesothosaurus* as the most basal thyreophoran [26] has subsequently been abandoned ([27] but see [28]. Currently, *Scutellosaurus* is the most basal thyreophoran primarily because of the extensive body covering of osteoderms. It is known from several fragmentary skeletons [29], [30]. Colbert [29] reported that *Scutellosaurus* had five sacral vertebrae, having a crescent or D-shaped centra in end view; these centra were not coossified due to the immaturity of the specimens. The sacral neural arches are poorly known, and their spines do not appear to have fused into vertical plate. The posterior-most dorsals do not show any modification into a synsacrum, such as evidence of tightly appressed centra even if unfused. The ilia are very fragmentary, which Colbert attempted to reconstruct ([29], fig. 23). However, there is doubt as to the accuracy of this reconstruction in part because we cannot locate all of the features Colbert describes. In addition, Colbert assumed the fragments of the holotype ilium lay in their correct relative position when found despite large gaps of missing bone. Given the clayey, hence expansive, nature of the matrix, it seems doubtful that the bone fragments did not move, especially since Colbert ([29], figs. 6 and 24) shows bone fragments that have moved apart along fractures. The fragment identified as the preacetabular process by Colbert ([29], Fig. 5A) differs from the fragment identified by Rosenbaum and Padian [30] in that it is straight rather than ventrally curved. The fragment identified as the mid-section of the ilium retains the dorsal edge, with a small fragment of the supra-acetabular crest. A digital reconstruction using the fragments of the holotype *Scutellosaurus* superimposed on the ilium of *Stormbergia* is shown in Fig. 5B. The match was better than when the ilium of *Heterodontosaurus* was used, so we predict that when a complete ilium of *Scutellosaurus* is found, it will more resemble that of *Stormbergia* or the similar looking one of *Lesothosaurus*, than that of *Heterodontosaurus*.

The pubis of *Scutellosaurus* is incomplete. The prepubic region is damaged, but there is no reason to a priori assume the presence of a well-developed prepubic process as Colbert [29] reconstructs. In light of the short, stubby prepubic process of *Stormbergia* and *Lesothosaurus*, it seems more probable that it was similar. The postpubic process is represented by the distal end, which is laterally compressed and deep. In cross-section the process is teardrop shaped, being wider dorsally and tapering ventrally. In lateral view, the process is curved ventrally. The distal apex is hidden, so it is not possible to determine its shape. The in situ position of the fragment indicates that the postpubic process extended to the end of the ischium, thus was long. The ischium is also incomplete, but the fragments suggest a partial twist of the shaft as in *Stormbergia* [31]. The distal end is damaged, but does seem to flare in profile. There is little about the pelvis that would identify *Scutellosaurus* as thyreophoran. We may, however, consider it as the basal ornithischian bauplan from which the thyreophoran pelvis could develop as is discussed further below.

Several phylogenetic analyses place *Scelidosaurus* as the sister group to the Eurypoda (Stegosauria + Ankylosauria) (e.g., [11], [25], [26], [32], despite evidence suggesting it is a basal ankylosaur [33]–[35]. The pelvis of *Scelidosaurus* differs from the more primitive state seen in *Stormbergia* and *Lesothosaurus*. First, it is proportionally wider due to lengthening of the sacral ribs (Fig. 3A) and outwardly sloping ilia (Fig. 3F). This results in the pelvis being wider than long, a condition unusual among ornithischians where pelvic length is typically greater. Second, the elongation of the sacral ribs is not uniform, being longer anteriorly and progressively shorter posteriorly, thus forming an isosceles trapezoid in dorsal view (Fig. 3A). This results in the sacral yoke angling seven degrees anterolaterally from the midline. This pattern of sacral ribs narrowing posteriorly is seen in other ankylosaurs (Fig. 6), and was independently acquired in other ornithischians, such as *Thescelosaurus* ([36], pl. 1, fig.1). This trapezoid shape is different from that seen in other ornithischians (e.g., *Camptosaurus* CM 11337), in which the sacral ribs form an hour-glass shape, being shortest adjacent to the acetabulum; an inverse hour-glass in which the sacral ribs are longest in the middle is seen in ceratopsians ([37] fig. 55) and stegosaurs (see [16], fig. 5; [38], pl. 24). A reverse pattern of sacral rib lengthening posteriorly occurs in other ornithischians (e.g., *Dryosaurus* CM 3392; *Gryposaurus* [39], pl. 5B; *Prenocephale* [40], fig. 5A; *Mantellisaurus* [41], fig. 6).

The synsacrum of *Scelidosaurus* is comprised of at least the first presacral (i.e., last dorsal vertebra) closely appressed against the first sacral (although these have now been separated by acid preparation). The opposing articular surfaces of the dorsal and sacral centra are somewhat irregular and interlocking, thus resembling the typical irregular surface between sacral vertebrae of immature dinosaurs and implies the two vertebrae of *Scelidosaurus* were tightly interlocked. There is no caudal incorporated into the synsacrum. The sacrum of *Scelidosaurus* is composed of four vertebrae, which are unfused in BMNH R1111, owing to the immaturity of the specimen (the condition is unknown in the larger specimen represented by SGDS 131 due to matrix). The neural spines are distinct and not fused into a plate as seen in later ankylosaurs. Within the acetabulum, a ventral portion of sacrals 1 and 2 form a small part of the dorsomedial rim of the acetabulum, as it does in *Gargoyleosaurus*, as well as *Gastonia*, and *Edmontonia* (NMC 8531); the ribs are excluded from the acetabulum in primitive ornithischians (e.g., [31]) and stegosaurs, including *Huayangosaurus*.

**Figure 7:**
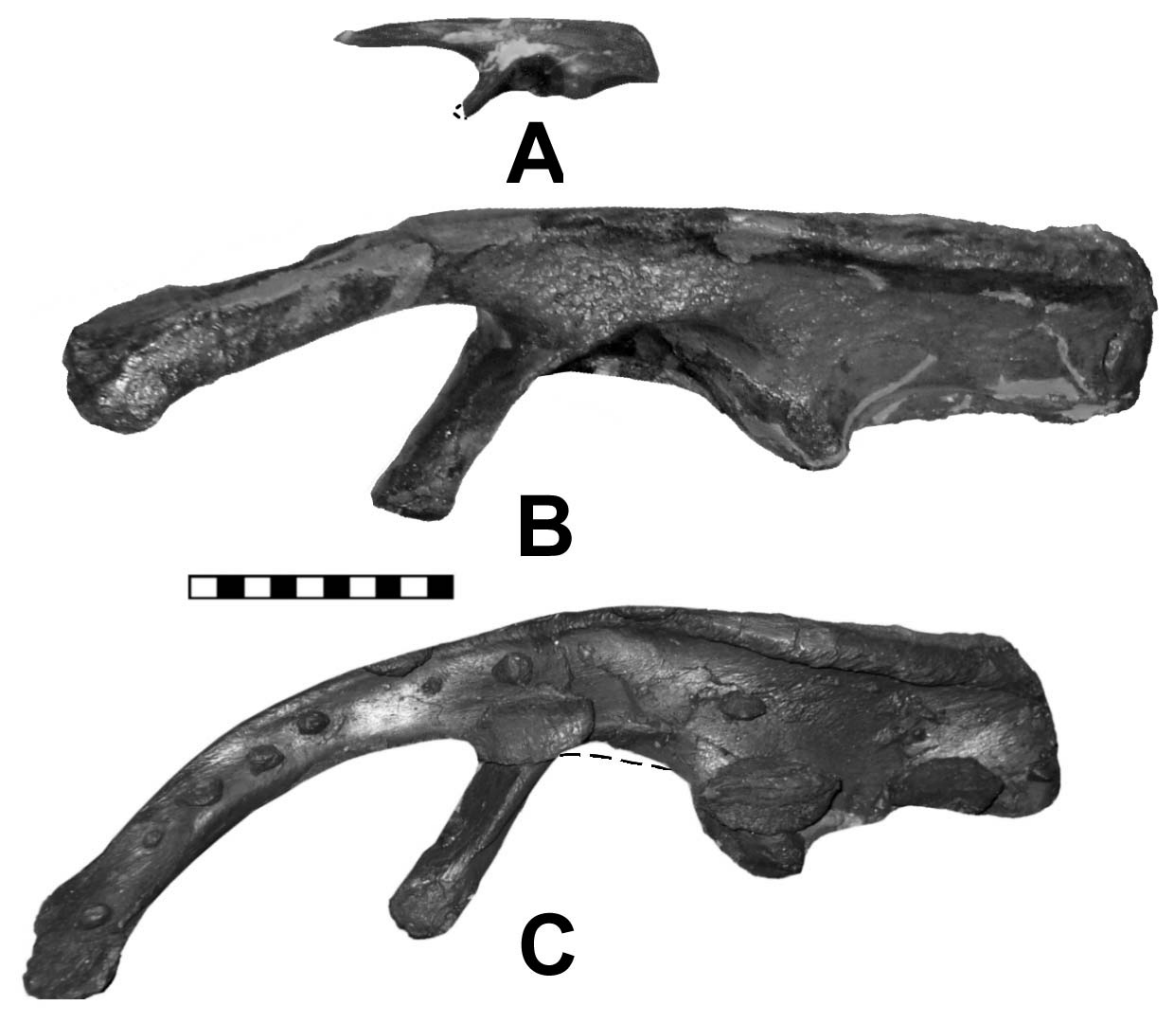
Ontogenetic series of *Scelidosaurus* ilia showing a change in the ventral angulation of the preacetabular process. An analogous situation was reported for ceratopsids by Lehman [42]. Additional changes include development of the lateral crest on the rim of the postacetabular process and greater development of the ischial peduncle. A, BMNH R6704; B, BMNH R1111; C, SGDS 131. Scale in cm.

Butler [31] noted similarities between the ilia of *Scelidosaurus*, *Stormbergia* and *Lesothosaurus*, and rightly noted these similarities are retention of plesiomorphic characters for the Ornithischia. We note, however, several important differences: In profile, the juvenile ilium of *Scelidosaurus* superficially resembles that of *Stormbergia*, except for having a deeper postacetabular process that is almost 150% greater. The process lengthens ontogentically and becomes longer than deep (Fig. 7). The dorsal rim of the postacetabular process has a lateral iliac crest (terminology from [5]) in subadult *Scelidosaurus*, but not in the juvenile ilium used by Butler [31] (Fig. 3G; 7A); this crest is not seen in the ilium of basal ornithschians. In addition, when articulated with the sacral vertebrae, the preacetabular process angles so that the medial surface faces medioventrally (Figs. 3E) over the posterior dorsal ribs; these ribs, however, do not fuse to the ilium as they do in ankylosaurs. The region above the acetabulum (i.e., dorsal plate) and postacetabular process rotated laterally to overhang the femoral head (Fig. 3F). In ventral view, the postacetabular crest in *Scelidosaurus* extends from the ischial peduncle to the posterior margin of the ilium, where it creates a terminal triangular thickening or swelling. The crest is better developed in the larger BMNH R1111 than in the juvenile (BMNH R6704). The crest delineates a narrow brevis shelf medially. In lateral view, the preacetabular process changes ontogenetically from horizontally directed in juveniles (BMNH R6704) to inclined (below horizon) in the adult (SGDS 1311); Lehman [42] previously reported a similar trend in *Agujaceratops mariscalensis* (originally as *Chasmosaurus mariscalensis*). The preacetabular process in *Scelidosaurus* is T-shaped in cross-section, with a well-developed medial shelf and narrow lateral iliac crest; the distal end is more expanded in the larger individuals than in the smallest. The acetabulum is partially closed medially between the pubic and ischial peduncles by a medial wall or acetabular lamina (ventral flange of some authors) of the ilium against which sacral ribs 1 and 2 articulate medially. The lamina is better developed in the juvenile than the adult because of the underdevelopment of the ischial peduncle. An acetabular lamina is also noted in *Lesothosaurus* and *Stormbergia* [31] (we have been unable to substantiate its presence in *Scutellosaurus*, contrary to Butler [27], but suspect it may have been present). A very short acetabular lamina is present in *Stegosaurus*, even in juveniles.

**Figure 8:**
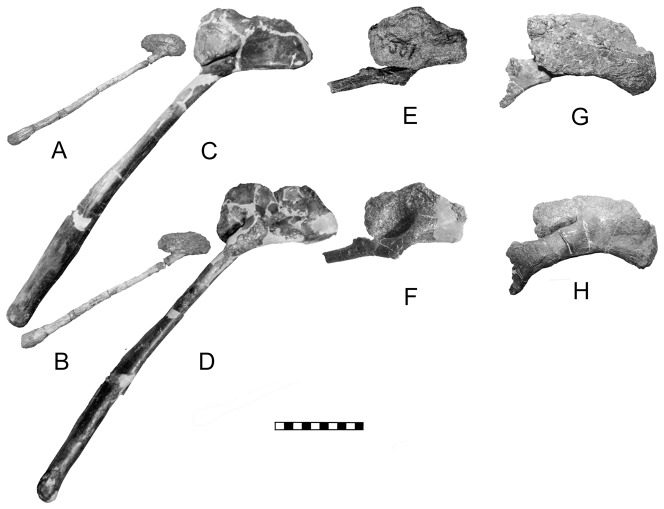
Modification of the pubis during ankylosaur origin and evolution may be illustrated by *Stormbergia* (SAM-PR-K1105) in right lateral (A) and medial reversed (B); *Scelidosaurus* (BMNH R1111) in right lateral (C) and medial reversed (D); *Gargoyleosaurus* (DMNH 27726) in right lateral (E) and medial reversed (F); *Edmontonia* (USNM 11868) in right lateral (G) and medial reversed (H). The two biggest changes seen are: 1) the rotation of the dorsal surface of the pubis (*Stormbergia*) so as to face laterally (*Scelidosaurus*); 2) slenderizing and shortening of the postpubic process between *Scelidosaurus* and *Gargoyleosaurus*. Scale in cm. A, B courtesy of R. Butler, Univ. Birmingham.

The pubis of *Scelidosaurus* is hammer-shaped or club-shaped and is similar in appearance to that of *Stormbergia* and *Lesothosaurus*, especially in the development of the short, deep prepubic process (Fig. 8A, B). However, unlike *Stormbergia*, the acetabular surface is expanded and rotated laterally so as to hide the obturator notch, a feature also seen in ankylosaurs, neoceratospians and stegosaurs. This rotation of the acetabular surface makes the pubic body laterally swollen; in stegosaurs, including *Huayangosaurus* [43] the acetabular surface is flat to concave. The ischia of *Scelidosaurus* are proportionally robust for their length and are rotated 90° so as to have a long midline symphysis as in *Lesothosaurus* ([31], fig. 4). The ischia lack an obturator process, which is present in *Stormbergia* [31].

**Figure 9:**
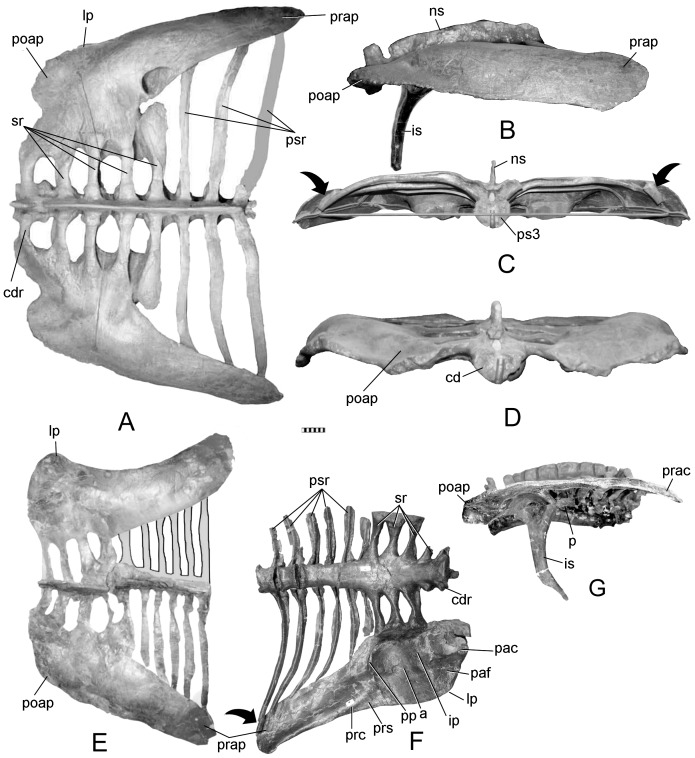
Pelvis of the ankylosaurid *Euoplocephalus tutus* (AMNH 5337 with ischium added to B). Views in: A, dorsal; B, right lateral; C, anterior; and D, posterior. Pelvis of the nodosaurid *Edmontonia*. *E. longiceps* (AMNH 5381) view in dorsal, E. *E. longiceps* (NMC 8531) in ventral, and F, lateral views. G Restored pelvis (neural spines restored from BHI 127327) mount and pubis from (USNM 11868). The ischium (NMC 8531) does not completely close the medial wall of the acetabulum, although it may have been blocked by the sacral yoke. Note how the preacetabular process overlies the presacral rib in C and F. Scale in cm. Abbreviations: a – acetabulum; cd – caudal vertebra; cdr – caudal rib; ip – ischial peduncle; is – ischium; lp – lateral process; ns – neural spines; p – pubis; pac – postacetabular crest; paf – postacetabular fossa; poap – postacetabular process; pp – pubic penducle; prap - preacetabular process; prc – preacetabular crest; prs – preacetabular shelf; ps3 – presacral vertebra 3; psr – presacral vertebrae ribs; sr – sacral rib(s). E, courtesy of K. Seymour, Nat. Mus. Canad.

The ankylosaurid pelvis is represented by that of *Euoplocephalus* (Fig. 9A-D). The synsacrum is well developed, consisting of three presacral vertebrae, four sacral vertebrae, and one caudal vertebra. The neural spines of the presacrals and sacrals form a vertical plate (Fig. 9B). The first presacral rib is coossified to the medial edge of the ilia, whereas the ribs of the other two presacrals underlap and coossify with the preacetabular process as in *Gargoyleosaurus*. The sacral ribs become progressively shorter so that the last one is about three-quarters the length of the first. The result is that the sacral yoke is angled anterolaterally about twenty-six degrees (AMNH 5470). The preacetabular process extends anteroventrally in a manner similar to *Gargoyleosaurus* so that the primitive medial surface faces ventromedially and overlies the posterior ribs (e.g., [17], fig 13; [44], fig. 2). The lateral and medial borders of the preacetabular process are parallel. The postacetabular process of the *Euoplocephalus* ilium is very short as noted by Coombs [44]. The process is widened medially by the expansion of the distal ends of the ribs of the last sacral and caudosacral vertebrae. This expansion of the ribs forms a broad shelf, which appears to have the function of the brevis shelf; the true postacetabular process of the ilium is the smaller and narrower posterior protrusion (Fig. 9A). The transversely oriented pubic peduncle of the ilium forms the anterior wall of the acetabulum, against which the pubic peduncle of the ischium contacts. Like an unnamed ankylosaurid (HMNS97-21-1), possibly *Pinacosaurus*, and *Saichania* (IGM 100/1305), there is no pubis. Romer ([1], p. 252) had previously suggested that possibility in ankylosaurs: “it is equally reasonable to believe that a separate large pubis was non-existent in all of them” based on his examination of *Euoplocephalus* (as *Ankylosaurus*); Arbour and Currie [11] came to a similar conclusion based on their study of *Euoplocephalus*. The ischium has a slightly curved, lateromedially compressed shaft that projects ventrally. Proximally, the ischium forms most of the medial wall of the acetabulum, with a slight contribution from the iliac lamina between the pubic and ischial peduncles. The acetabular fenestra is absent.

The pelvis of *Edmontonia* represents the nodosaurid condition. The synsacrum consists of four (NMC 8531, USNM 11868) or five (AMNH 5381) presacral vertebrae, four sacral vertebrae, and one caudal vertebra (Fig. 9E). The identification of two caudals [45], [46] was an error corrected by the less damaged AMNH 5381. The centra of presacral 1 and sacral 1 have a slight groove along their ventral side. Sacral 1–3 are broad due to gangliar enlargement of the neural canal. The neural spines of the presacral and sacral vertebrae form a vertical plate. The first presacral rib contacts the medial edge of the ilium, but does not fuse. This does not appear to be due to immaturity because it occurs in NMC 8531 and AMNH 5381 where the vertebrae show complete closure of the neural arch suture; the other presacral ribs under-lap and coossify with the preacetabular process. Although the sacral ribs sequentially shorten posteriorly, the shortening is significantly less than in ankylosaurids (see also [47], pl. 3 for *Nodosaurus*). The sacral yoke is angled anterolaterally about 17 degrees from the midline.

In lateral view, the preacetabular process is folded medially much more than in *Gargoyleosaurus* and *Euoplocephalus* so that it is almost completely horizontal (Fig. 9G). The medial and lateral edges of the ilia are subparallel, with a slight narrowing anteriorly. Ventrally, the short, blunt, ridge-like pubic peduncle projects anteroventrally from the ilium. The ridge continues medially in an arc to form the anterior rim of the acetabulum, as well as a low acetabular lamina; posteriorly, it joins the low ischial peduncle. The preacetabular crest extends anteriorly from the base of the pubic peduncle and delineates a small, triangular preacetabular shelf. The postacetabular process is broad and rounded in *E. rugosidens* (AMNH 5381), lacking the posterior protrusion seen in *Gargoyleosaurus* or *Euoplocephalus*. There is a short protrusion in *E. longiceps* (NMC 8531), with a broad postacetabular crest on the ventral side. This crest delineates a shallow, triangular postacetabular fossa.

A small pubis for *Edmontonia* is known only for USNM 11868, the holotype of *E. rugosidens* (Fig. 8G,H). It resembles that of *Sauropelta* (YPM 5141) in being laterally compressed and arcuate in profile, rather than a rectangle as in *Gargoyleosaurus*. The lateral face is irregular, rugose and extensively pitted, as it is in *Gargoyleosaurus* ((Fig. 8E) and *Sauropelta*, making the surface appear eroded as erroneously assumed by Gilmore [45]. The presence of this surface texture in all the specimens suggests an articular cartilage covering. The postpubic process extends from the medial side and has a slight expansion that braces the bone against the ischium. There was apparently little or no contact between the pubis and the pubic peduncle of the ilium in a manner analogous to that of crocodilians and perhaps to pachycephalosaurs [40]. The ischium along with the acetabular lamina forms the medial wall of the acetabulum. There may have been a slight gap between the lamina and ischium, although this is equivocal because the lamina may be damaged in the one good ilium where this region can be seen (NMC 8531); it is completely closed in *Animantarx* where the ischium is coossified with the ilium [48]. If a small, slit-like acetabular fenestra is present in *Edmontonia*, then the sacral yoke of the sacral ribs backed it. The ischium has a wide, cupped proximal end that forms the acetabulum. Its shaft is laterally compressed and distally tapering, with an anteriorly directed bend in the distal third. The iliac peduncle is large compared to the pubic peduncle.

### Trends in Development

**Figure 10:**
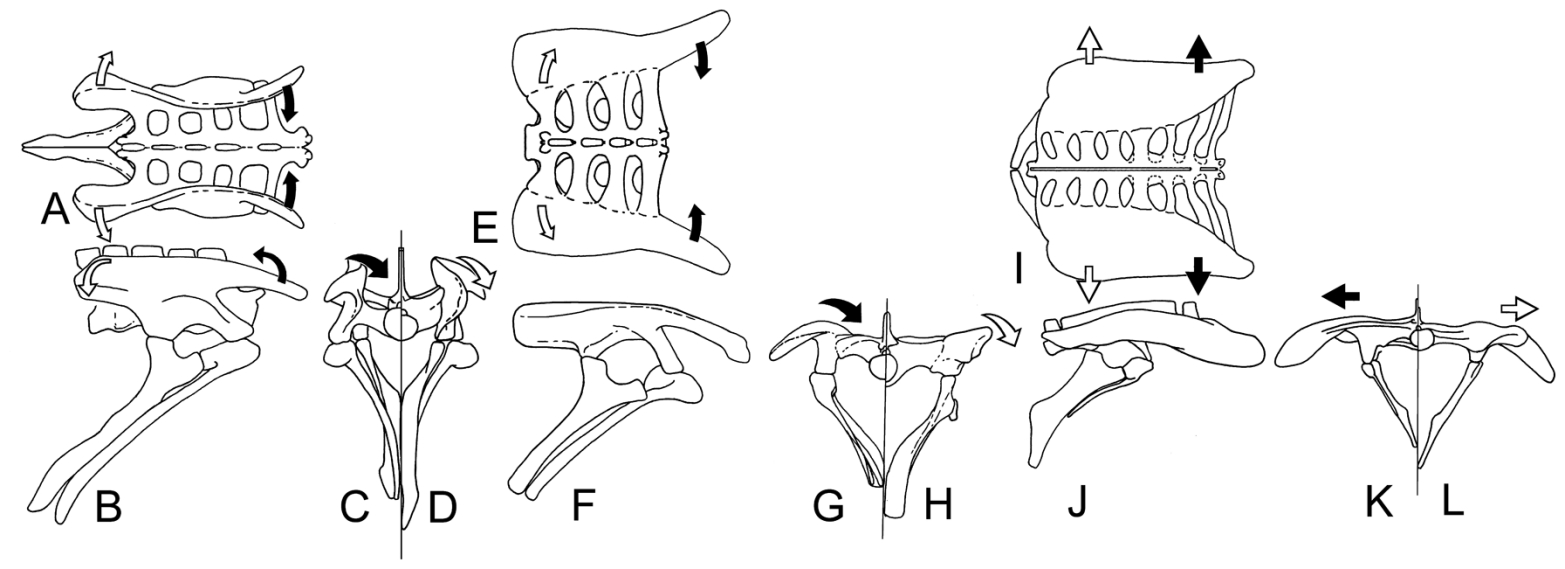
Summary of the pelvic changes during the origin and early evolution of the ankylosaurs. The primitive ornithischian condition is represented by *Scutellosaurus* (modeled after *Stormbergia*) in dorsal (A), right lateral (B), anterior (C – with foreshortening of the ischium), and posterior (D) views. Black arrows denote the medial direction the preacetabular process would rotate to acquire the *Scelidosaurus* condition, and white arrows the lateral direction of the postacetabular process. Pelvis of *Scelidosaurus* in dorsal (E), right lateral (F), anterior (G – with foreshortening of the postpubic process and ischium), and posterior (H) views. Black arrows denote the medial direction the preacetabular process would rotate to acquire the *Gargoyleosaurus* condition, and white arrows the lateral direction of the postacetabular process. Pelvis of *Gargoyleosaurus* in dorsal (I), right lateral (J – with ischium of *Mymoorapelta*), anterior (K – with foreshortening of the ischium), and posterior (L) views. Subsequent changes from the *Gargoyleosaurus* condition in more advanced ankylosaurs (e.g., *Euoplocephalus* and *Edmontonia*) involve lateral expansion of the preacetabular process (black arrows) and postacetabular process (white arrows). Not to scale.

From the above descriptions, it is possible to show some trends in the early evolution of the ankylosaur pelvis (Fig. 10). The pelvis most likely developed from one similar to that seen in *Scutellosaurus* (Fig. 10A–D), *Stormbergia* or *Lesothosaurus*, in which the ilium was a relatively vertical plate having a relatively short preacetabular process, short, moderately deep postacetabular process with a narrow brevis shelf. The pubis had a short, stubby prepubic process, an obturator foramen visible in lateral view, and a long postpubic process. The ischium has a long shaft, and may or may not have had an obturator process. The next stage in the ankylosaur pelvis evolution is represented by the pelvis of *Scelidosaurus* (Fig. 10E–H) The pelvis of the juvenile bridges the morphological gap between that of the more mature specimens and that of more basal ornithischians. Many plesiomorphic characters are retained in the juvenile, but become modified ontogenetically (Fig. 7): specifically, the preacetabular process rotates medially so that the medial surface faces medioventrally and overlies the posterior dorsal ribs (Fig. 10G). The dorsal plate, that region dorsal to the acetabulum, as well as the postacetabular process, counter-rotates laterally to overhang the acetabulum (Fig. 10H). The pubis of *Scelidosaurus* is little changed from that of the basal ornithischian condition in having a short, deep body, and a long rod-like postpubic process. The prepubic process, however, is slightly lengthened and laterally compressed (Fig. 10F). In addition, the pubic body is rotated to obscure the obturator fenestra in lateral view. The ischium is simple, and resembles that of *Stormbergia* in that it has a long, robust shaft, but differs in the absence of an obturator process. A synsacrum composed of sacral vertebrae and at least one dorsal was present. The sacral ribs are longer so that the ilia are proportionally father apart than in more basal ornithischians (compare 10E with 10A). The neural spines of the synsacrum are not fused into a vertical plate, and thus retaining the plesiomorphic condition.

The pelvic modification trend especially of the ilium represented by *Scelidosaurus* continued with the development of the subhorizontal ilium seen in *Gargoyleosaurus* (Fig. 10H-K). The dorsal plate is horizontal, as is the postacetabular process, and both extend laterally well beyond the acetabulum (Fig. 10J, K). The postacetabular and preacetabular crests extending from the acetabulum can be correlated with the ventral edge of the ilium in *Scelidosaurus*, *Stormbergia* and *Lesothosaurus*. These ridges are important landmarks and demonstrate that direction of the rotation in ankylosaurs of the preacetabular process was counter to that of the postacetabular process. This interpretation is different from that of Coombs ([2]:667) who assumed “the entire ilium was twisted laterally so as to lie in the horizontal plane…” The medial rotation of the preacetabular process better explains how the posterior dorsal ribs came to underlie the ilium. Rather than the two-step process of the ilium rotating away from the ribs, then the ribs expanding under the ilium, a more simplified one-step has the ilium rotating medially over the ribs. The ramifications are that at least a portion of the hindlimb musculature that has been restored [2], [4], is now in doubt and in need of re-evaluation.

In addition, the pubis of *Gargoyleosaurus* shows that several important changes took place from the more primitive condition. First, the postacetabular process was reduced to a slender rod, the length of which is unknown (Fig. 8E, F). However, in *Mymoorapelta*, the rod-like postpubic process is long and extends at least to the middle of the ischium (MWC 2610). Second, the pubic body is set at a slight angle relative to the postpubic process so that when articulated with the ilium, it is at an angle relative to the body midline. This trend is further developed in nodosaurids, such as *Sauropelta*, where the pubis is nearly at right angles and forms the anterior rim of the acetabulum. Third, the lateral or acetabular face of the pubic body is rugose and must have been covered with cartilage; the same feature is seen in nodosaurids, such as *Sauropelta* and *Edmontonia.* The ischium is unknown for *Gargoyleosaurus*, but given that the ischium is relatively conservative in ankylosaurs, it may have resembled that of *Mymoorapelta* (Fig. 10I). If true, then the ischium did not extend ventrally as in later ankylosaurs, but angled ventroposteriorly at an angle similar to *Scelidosaurus* (Fig. 3). In addition, the ischium did not completely close the acetabulum medially as in later ankylosaurs, but rather it left a large gap, or acetabular fenestra, that was partially blocked by the sacral yoke. The synsacrum in *Gargoyleosaurus*, composed of sacral, dorsal and caudal vertebrae in which their neural spines are fused into a vertical plate, resembles that of both nodosaurids and ankylosaurids.

The broad typical ankylosaur pelvis is present in nodosaurids and ankylosaurids [2], [3], [17], [23]. Although they are similar in having widely splayed ilia, they also have some distinct differences that indicate a long separate evolution. Nodosaurids retain sacral ribs that are almost the same length, with the first rib only slightly longer than the last. This feature is plesiomorphic for ankylosaurs and is seen in *Scelidosaurus* and *Gargoyleosaurus*. In contrast, the first sacral rib in ankylosaurids may be a third longer than the last sacral rib. The preacetabular process in nodosaurids has rotated medially to a more horizontal position than in ankylosaurids. The pubis is small, but distinct in nodosaurids where it forms the anterior wall of the acetabulum (e.g., *Edmontonia*, *Sauropelta*). Superficially, it resembles that of *Gargoyleosaurus*. In contrast, the pubis in ankylosaurids is reduced further in size and is lost in some taxa (e.g., *Euoplocephalus*, *Saichania*). The ischium in both nodosaurids and ankylosaurids rotates from its presumed posteroventral projection in *Gargoyleosaurus* (see above) to projecting ventrally in nodosaurids and ankylosaurids. This rotation is accompanied by filling of the gap between the iliac and pubic peduncles by the iliac lamina so that there is complete or nearly complete closure of the medial acetabular fenestra. In addition, the distal end of the ischium in *Edmontonia* is bent sharply forwards, a feature also seen in the holotype of *Sauropelta* ([49], pl. 25A, B).

### Pelvis Modification in Other Ornithischians

**Figure 11:**
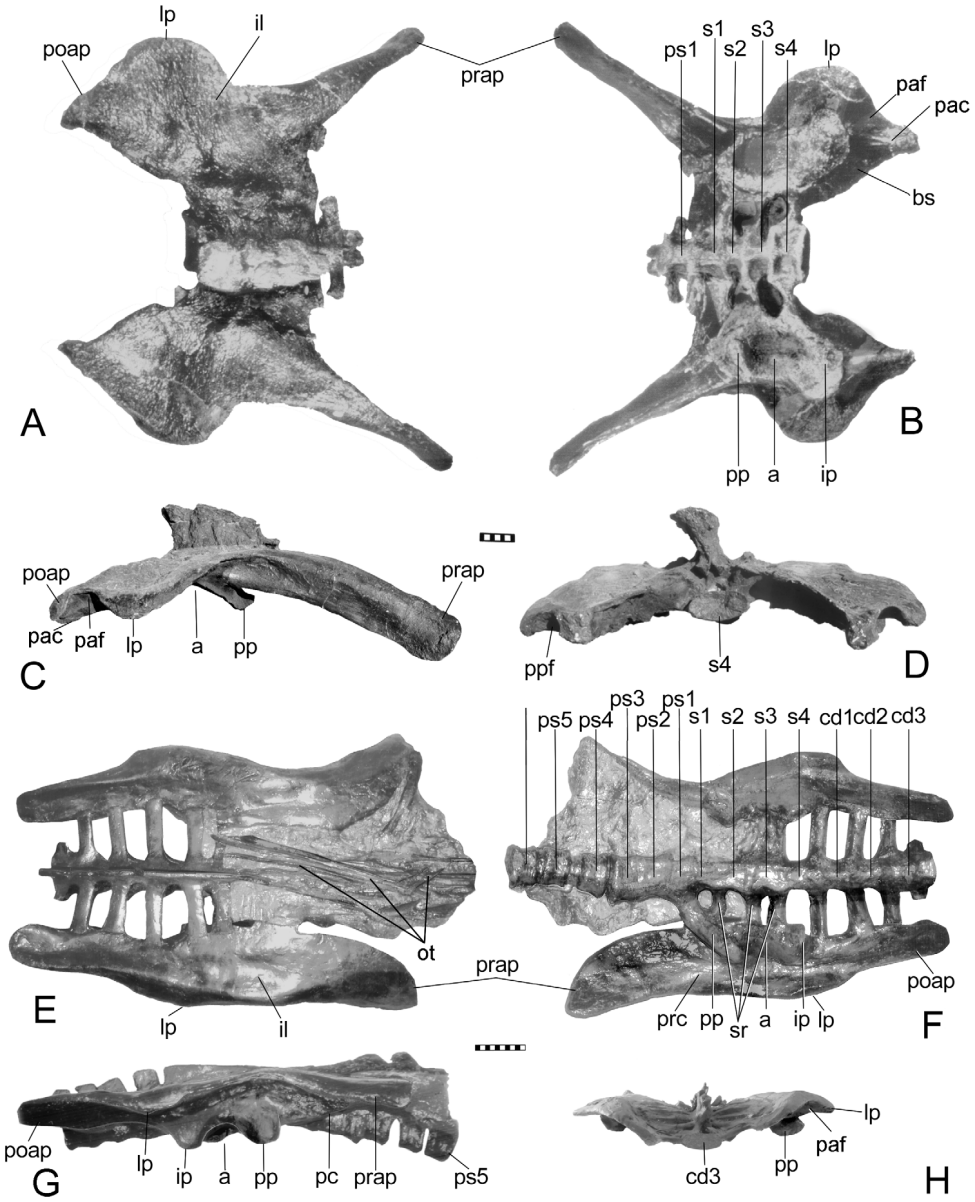
Horizontal ilium seen in other quadrupedal ornithischians, including stegosaurs, typified by *Hesperosaurus mojsi* (HMNH 001) and neoceratopsians, typified by *Chasmosaurus belli* (ANSP 15764). Views in dorsal (A, E), ventral (B, F), right lateral (C, G) and posterior (D, H). Scales in cm. Abbreviations: a – acetabulum; bs – brevis shelf; cd1-3 – caudal vertebra 1 – 3; cdr – caudal rib; ip – ischial peduncle; lp – lateral process; ot – ossified tendons; pac – postacetaular crest; paf – postacetabular fossa; poap – postacetabular process; pp – pubic penducle; poap - postacetabular process; prap - preacetabular process; prc – preacetabular crest; ps1–6 – presacral vertebrae 1–6; s1–4 – sacral vertebrae 1–4; sr – sacral rib(s).

**Figure 12:**
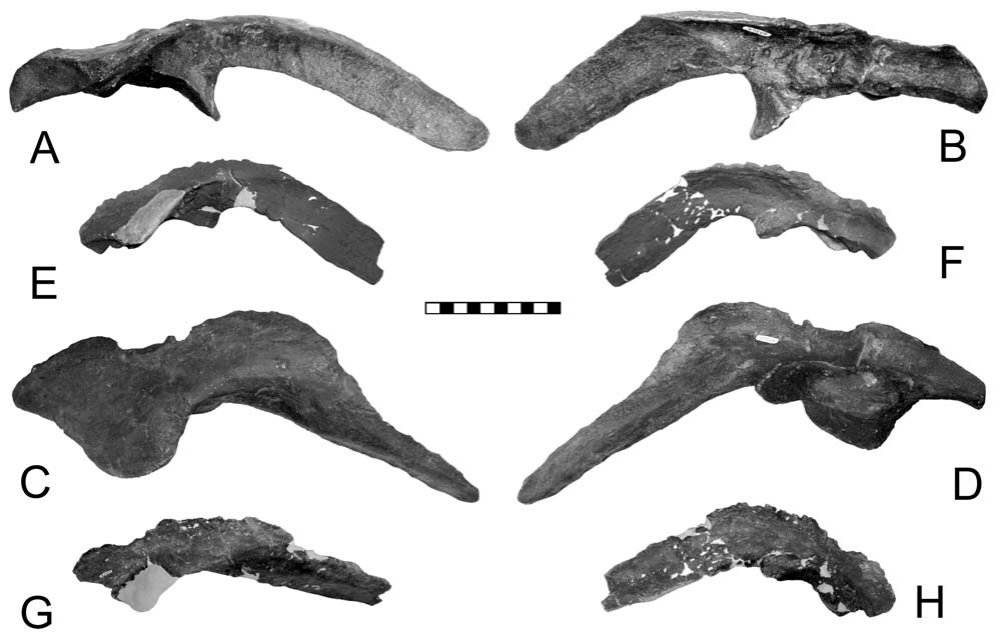
Juvenile *Stegosaurus* ilia: A–D, DMNH 33360 (cast of DINO 2438); DMNH 33359. Views: A, E right lateral; B, F medial; C, G dorsal; D, H ventral. Scale in cm.

**Figure 13:**
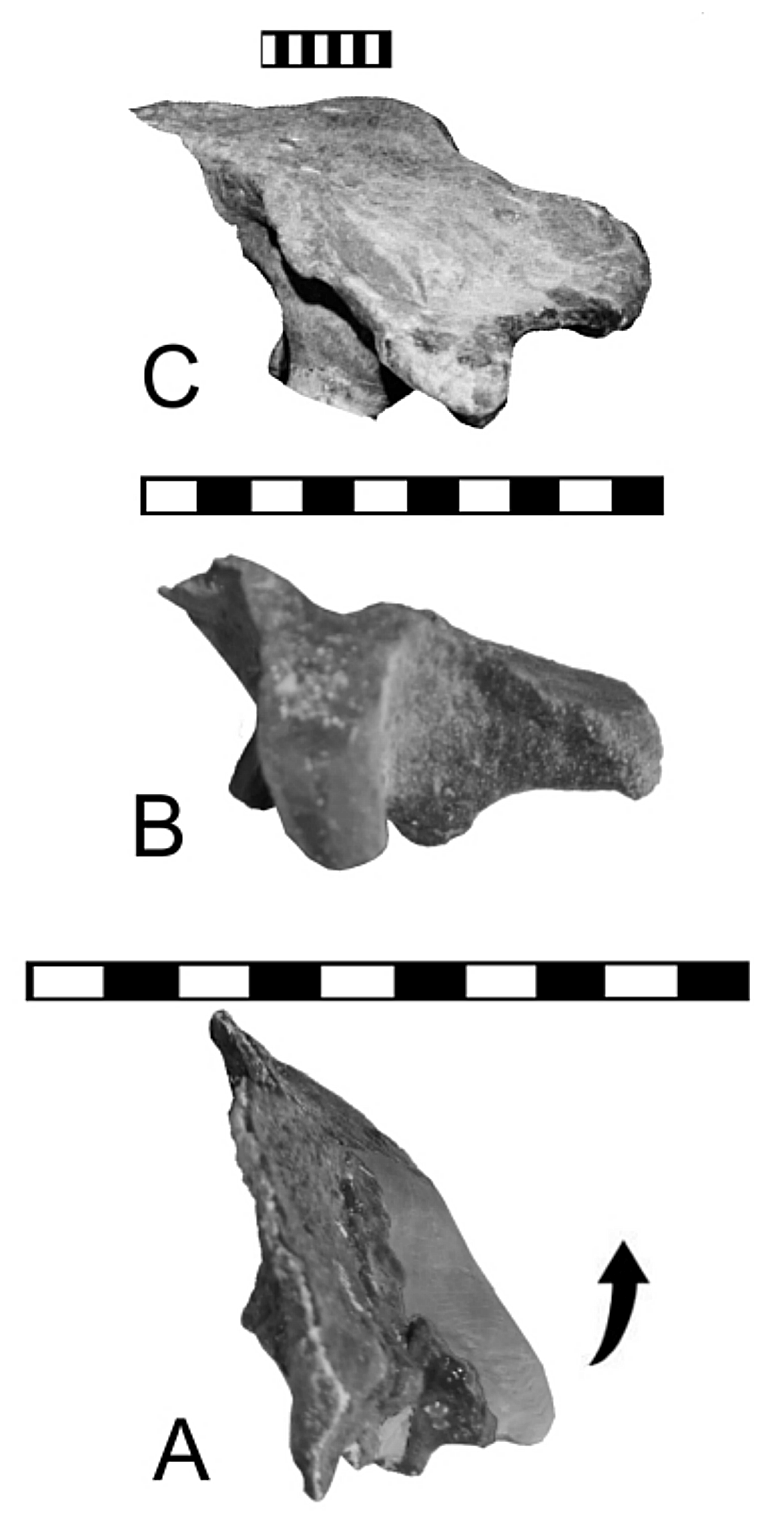
Ontogenetic change in the position of the lateral process ( = supra-acetabular process) in *Stegosaurus stenops* as seen from the rear. The process rotates dorsally very early in ontogeny (compare A with B, B with C). A, DMNH 33359; B, DMNH 33360; D, DMNH 1438. Scales in cm.

Some of the changes seen in the pelvis, particularly the ilium, during ankylosaur evolution occur in varying degrees in other ornithischians. These are briefly examined for the clues they may provide for understanding ankylosaur pelvic evolution. Using the ventral ridge or margin as the plesiomorphic ventral margin for ornithischians, the ilium of stegosaurs shows a mixture of primitive and derived features (Fig. 11A–D). First, the preacetabular process is nearly vertical, although leans medially somewhat so as to overlap the posterior-most ribs; there are no fusion between these structures as in ankylosaurs. Second, primitively the ilia diverge slightly from the midline (e.g. ∼20° *Huayangosaurus*), and become more divergent in more derived stegosaurs (e.g., ∼32° *Hesperosaurus*, Fig. 11A, B). The postacetabulum is subhorizontal, as is the dorsal plate. The lateral process (supra-acetabular process of [16]) is reinforced ventrally by a ridge forming a robust buttress. An early ontogenetic series of *Stegosaurus* ilia (Fig. 12) show that the lateral process is not formed by the dorsal rim of the postacetabular rotating laterally to a horizontal position. Rather, the dorsal rim rotated medially pulling the lateral process and the postacetabular process dorsally (Fig. 13). This mode of development of the postacetabular process to its near horizontal position has major ramifications for thyreophoran evolution: Namely, that the subhorizontal postacetabular process of stegosaurs forms by medial rotation in opposition to the lateral rotation of ankylosaurs. This also means that *Scelidosaurus*, with its laterally folded subhorizontal postacetabular process, cannot be more basal to both stegosaurs and ankylosaurs as is often stated [16], [25], [26], [32]. The reason is that there would have to be a complete reversal of the postacetabular process rotating laterally to rotating medially, plus a shift in the development of a lateral process from the lateral surface of the ilium to the dorsal rim. This would also entail a major shift in accompanying musculature. Therefore, *Scelidosaurus* is a basal ankylosaur as previously stated [33], the Ankylosauromorpha is a valid group composed of *Scelidosaurus* + Ankylosauria. The development of the subhorizontal postacetabular process arose independently in stegosaurs and ankylosaurs, as it apparently did in the marginocephalians given the very different structure between pachycephalosaurs and ceratopsids.

**Figure 14:**
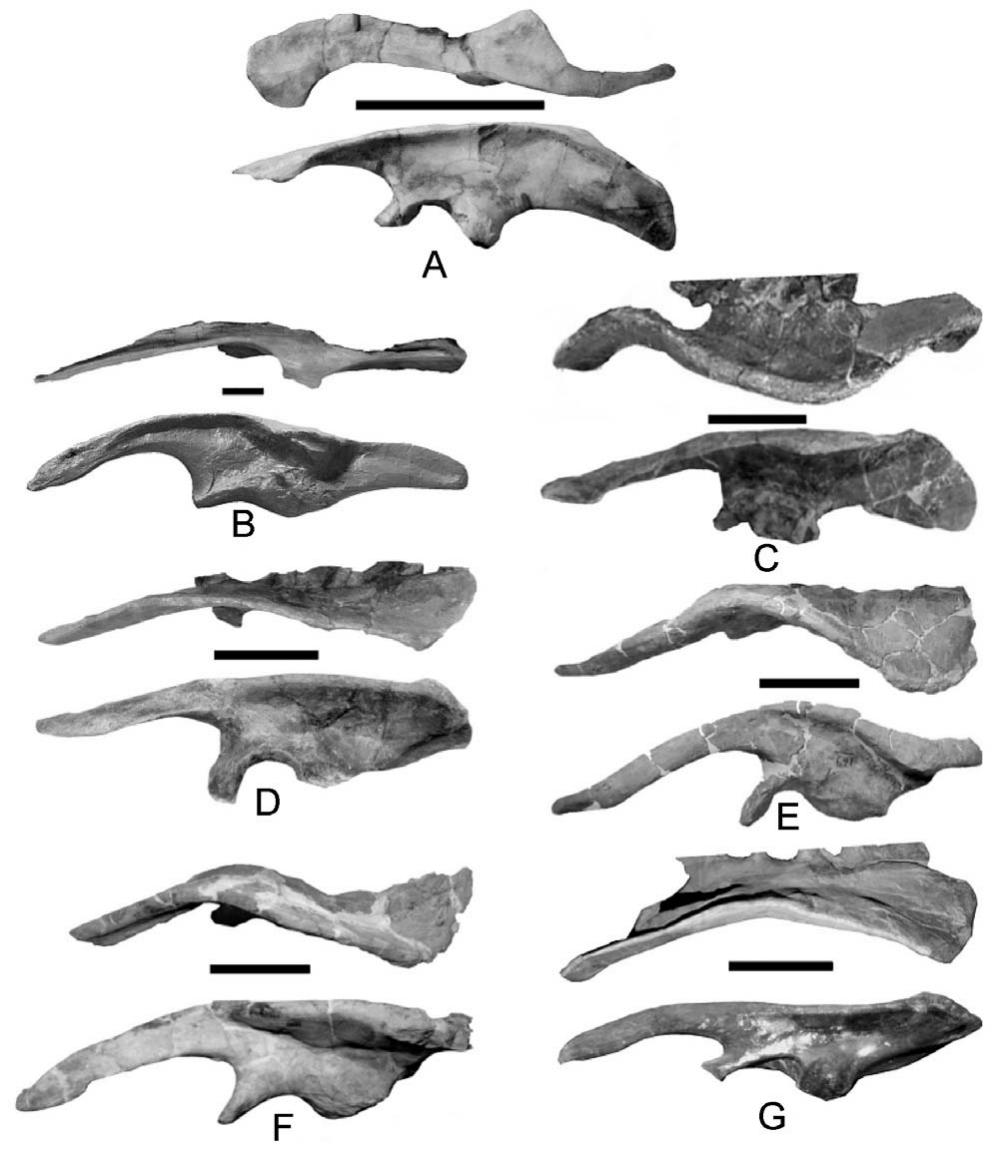
Examples of iliac modification in a variety of non-thyreophoran, non-ceratopsian ornithischians in dorsal (top) and lateral (bottom) views. A, lateral extension of the preacetabular process and development of a medial process in the pachycephalosaurid *Homalocephale* (IGM 100/51); B, lateral development of the lateral iliac crest and lateral process along the dorsal margin in the hadrosaur *Edmontosaurus* (BHI 126,414); C, lateral folding of the supra-acetabular portion ( = dorsal plate) of the ilium in the rhabdodontid *Zalmoxes* (modified from [53]). The most common modification is the lateral folding of the postacetabular process as seen in: D, *Camptosaurus* (CMNH 11337); E, *Planicoxa venenica* (DMNH 42,504); F, *Planicoxa depressus* (USNM 4759); G, *Thescelosaurus* (AMNH FR117). This folding is too similar among different taxa and specimens to be due to crushing. Scale bars  = 10 cm).

Ceratopsids are noted for their horizontal ilia (Fig. 11C–H), although the postacetabular process in adult centrosaurines is more vertical than in chasmosaurines. In dorsal view, the ilium is slightly sigmoidal, with the preacetabular process diverging from the midline and the postacetabular process angled towards the midline (Fig. 14A). Using the ventral ridge landmark, the preacetabular process is folded medially over the posterior-most ribs. However, these do not fuse to the underside of the preacetabular process as in ankylosaurs. As in *Stegosaurus*, the lateral process in juvenile chasmosaurines occurs low on the ilium (e.g. [42], fig.17) and the postacetabular process is vertical. With maturity, the lateral process rotates dorsally as in stegosaurs, the postacetabular portion of the lateral iliac crest expands laterally and the brevis shelf expands medially. In the lower pelvis bones, the pubis is unlike ankylosaurs in being anteriorly lengthened, but the postpubic process is short, and the main body is expanded to partially block the acetabulum medially. The ceratopsid synsacrum is composed of three presacral, four sacrals and three caudals.

Pachycephalosaurs expanded their ilia horizontally, but did so differently than other ornithischians (Fig. 14B). The preacetabular process is expanded and folded laterally rather than medially, whereas the medial crest is expanded on the postacetabular process. On the medial side is a triangular process, or flange, that is located above or slightly posterior to the ischial peduncle. Primitively, the postacetabular process is a vertical plate (e.g., *Goyocephale*), but in more advanced taxa the medial iliac crest is expanded to overlie the distal ends of the caudal ribs.

The greatest variety of iliac modification occurs among the ornithopods. These changes range from the little modified ilium of *Barilium* [50] to the laterally folded dorsal rim of *Zalmoxes* (Fig. 14D) and *Iguanodon* [51], [52], to the laterally folded postacetabular process of *Camptosaurus* (Fig. 14E) and *Planicoxa* (Fig. 14F, G). We disagree with McDonald [50], [51] that the taxonomically widely distributed lateral folding of the ilium in ornithopods (Fig. 14B–H) is due to crushing and distortion. Crushing should preferentially distort the relatively thin, divergent preacetabular process ([51], fig 9). We find that the most frequent region of ilium folding laterally is the postacetabular process, followed by the dorsal plate above the acetabulum. Rarely among ornithopods is the preacetabular process expanded laterally beyond simple divergence from the midline. The affected portion of the ilium among the ornithopods relative to the position of the acetabulum suggests a biomechanically induced alteration associated with hindlimb musculature, primarily the M. iliotibialis and M. iliofibularis. Shifting the origin of these muscles laterally accomplishes three goals: 1) makes more room for the origin of these muscles, implying greater muscle bulk; 2) places the vector of the pull more in line with the insertion of these muscles; and 3) makes more room for the deeper M. iliofemoralis. In contrast, the ilium modification in ankylosaurs, as well as in ceratopsians and pachycephalosaurs, involves ribs, sacral ribs, and the preacetabular, as well as postacetabular processes. The alterations to the regions dorsal and posterior to the acetabulum may be due to modification of the locomotor musculature as in ornithopods, but modifications of the region anterior to the acetabulum may be due to an enlarged rear gut, with secondary modifications of the musculature. To resolve these questions requires a re-evaluation of hindlimb muscular reconstructions of ankylosaurs.

## Conclusions

Based on the relative positions of the ventrally placed postacetabular crest to the ischial peduncle and the ventrally placed preacetabular crest to the pubic peduncle, these crests appear to be homologous to the ventral margin of the primitive ornithischian ilium (e.g., *Stormbergia*). If correct, these structures may be used as landmarks to interpret ilium modification in ankylosaurs, and probably other ornithischians as well. In summary, the pelvis of ankylosaurs from a basal ornithischian condition underwent three stages of development (Figs. 6, 10). Beginning with the primitive pelvis represented by *Stormbergia*, the ilium was mostly a vertical plate, with a moderately developed, slender preacetabular process; the prepubic process was small; and the postpubic rod was elongate and paralleled the posteriorly directed, long ischium. The basal ankylosauromorph condition is characterized by elongation of sacral ribs; elongation and widening of the preacetabular process, which rotated medially to partially overlie the posterior dorsal ribs; and postacetabular process rotating partially laterally to overhang the femoral head; pubic body rotating laterally to block the obturator foramen. The more advanced ankylosauromorph condition is characterized by further rotation and widening of the preacetabular process; expansion of the proximal end of the ischium to form a partial medial wall of the acetabulum; and shortening of ischium. The more derived ankylosaurs are characterized by divergence of the preacetabular process, and elimination of the pubis in some ankylosaurids.

## References

[1] Romer AS (1927) The pelvic musculature of omithischian dinosaurs. Acta Zool 8: 225–275.

[2] Coombs WP (1979) Osteology and myology of the hindlimb in the Ankylosauria (Reptilia, Ornithischia). J Paleont 53: 666–684.

[3] Vickaryous MK, Maryańska T, Weishampel DB (2004) Ankylosauria. In: Weishampel DB, Dodson P, Osmolska H, editors. The Dinosauria, 2nd Edition. Berkeley: University of California Press. pp. 363–392.

[4] Maidment SC, Barrett PM (2012) Does morphological convergence imply functional similarity? A test using the evolution of quadrupedalism in ornithischian dinosaurs. Proc Royal Soc B: Biol Sci 279: 3765–3771. 10.1098/rspb.2012.1040 PMC3415913 22719033

[5] Carpenter K, Ishida Y (2010) Early and “Middle” Cretaceous Iguanodonts in Time and Space. J Iberian Geo 36: 145–164.

[6] Carpenter K, Miles C, Cloward K (1998) Skull of a Jurassic ankylosaur (Dinosauria). Nature 393: 782–783.

[7] Kilbourne B, Carpenter K (2005) Redescription of *Gargoyleosaurus parkpinorum*, a polacanthid ankylosaur from the Upper Jurassic of Albany County, Wyoming. N Jahrb Geol Paläont Abh 237: 111–160.

[8] Burns ME (2008) Taxonomic utility of ankylosaur (Dinosauria, Ornithischia) osteoderms: *Glyptodontopelta mimus* Ford, 2000: a test case. J Vert Paleont 28: 1102–1109.

[9] Carpenter K (2004) Redesciption of *Ankylosaurus magniventris* Brown 1909 (Ankylosauridae) from the Upper Cretaceous of the Western Interior of North America. Canad J Earth Sci 41: 961–986.

[10] Ford TL (2000) A review of ankylosaur osteoderms from New Mexico and a preliminary review of ankylosaur armor. New Mexico Mus Nat Hist Bull 17: 157–176.

[11] Arbour VM, Currie PJ (2013) *Euoplocephalus tutus* and the Diversity of Ankylosaurid Dinosaurs in the Late Cretaceous of Alberta, Canada, and Montana, USA. PLoS ONE 8(5): e62421 doi:10.1371/journal.pone.0062421 23690940 10.1371/journal.pone.0062421 PMC3648582

[12] Kirkland JI, Carpenter K (1994) North America's first pre-Cretaceous ankylosaur (Dinosauria) from the Upper Jurassic Morrison Formation of Western Colorado. Brigham Young Univ Geol Stud 40: 25–42.

[13] Osborn HF (1899) A skeleton of *Diplodocus*. Mem Amer Mus Na. Hist 1: 191–214.

[14] Owen R (1847). On the archetype and homologies of the vertebrate skeleton. John van Voorst, Paternoster Row.

[15] Owen R (1866) *On the anatomy of vertebrates* (Vol. 1): Fishes and Reptiles. London: Longmans, Green and Co. 650 p.

[16] Maidment SC, Wei G (2006) A review of the Late Jurassic stegosaurs (Dinosauria, Stegosauria) from the People's Republic of China. Geol Mag 143: 621–634.

[17] Coombs WP (1978) The families of the ornithischian dinosaur order Ankylosauria. Palaeont 21: 143–170.

[18] Coombs W, Maryanska T (1990) Ankylosauria. In: Weishampel DB, Dodson P, Osmolska H, editors. The Dinosauria. Berkeley: University of California Press. pp. 456–483.

[19] Hulke JW (1887) Supplemental note on *Polacanthus foxii*, describing the dorsal shield and some parts of the endoskeleton, imperfectly known in 1881. Phil Trans. R. Soc. (B) 178: 169–172.

[20] Nopsca F (1928) Palaeontological notes on reptiles. Geol Hungarica Ser Palaeont 1: 1–84.

[21] Maleev E (1954) Armored dinosaurs of Mongolia, Family Syrmosauridae. Trudy Paleont Inst Akad Nauk SSSR 48: 142–170.

[22] Maleev E (1956) Armored dinosaurs of Mongolia, Family Ankylosauridae Trudy Paleont Inst Akad Nauk SSSR. 62: 51–91.

[23] Carpenter K, Hayashi S, Kobayashi Y, Maryanska T, Barsbold R, et al (2011) *Saichania chulsanensis* (Ornithischia, Ankylosauridae) from the Upper Cretaceous of Mongolia. Palaeont Abt A 294: 1–61.

[24] Norman DB (1984) A systematic reappraisal of the reptile order Ornithischia. In: Reif WE, Westphal F, editors. Third Symp Mes Terr Ecosyst Tübingen: Attempto Verlag.

[25] Sereno PC (1986) Phylogeny of the bird-hipped dinosaurs (Order Ornithischia). Nat Geogr Res 2: 234–256.

[26] Butler RJ, Upchurch P, Norman DB (2008) The phylogeny of the ornithischian dinosaurs. J Syst Palaeont 6(1): 1–40.

[27] Butler RJ (2010) The anatomy of the basal ornithischian dinosaur *Eocursor parvus* from the lower Elliot Formation (Late Triassic) of South Africa. Zool J Linn Soc 160: 648–684.

[28] Norman DB, Crompton AW, Butler RJ, Porro LB, Charig AJ (2011) The Lower Jurassic ornithischian dinosaur *Heterodontosaurus tucki* Crompton & Charig, 1962: cranial anatomy, functional morphology, taxonomy, and relationships. Zool J Linn Soc 163: 182–276.

[29] Colbert EH (1981) A primitive ornithischian dinosaur from the Kayenta Formation of Arizona. Mus North Ariz Bull 53: 1–61.

[30] Rosenbaum JN, Padian K (2000) New material of the basal thyreophoran *Scutellosaurus lawleri* from the Kayenta Formation (Lower Jurassic) of Arizona. PaleoBios 20: 13–23.

[31] Butler RJ (2005) The ‘fabrosaurid’ornithischian dinosaurs of the upper Elliot Formation (Lower Jurassic) of South Africa and Lesotho. Zool J Linn Soc 145: 175–218.

[32] Thompson RS, Parish JC, Maidment SC, Barrett PM (2012) Phylogeny of the ankylosaurian dinosaurs (Ornithischia: Thyreophora). J Syst Palaeont 10(2): 301–312.

[33] Carpenter K (2001) Phylogenetic analysis of the Ankylosauria. In: Carpenter K, editor. The Armored Dinosaurs. Bloomington: Indiana University Press. pp 454–483.

[34] Norman DB (2001) The anatomy and systematic position of *Scelidosaurus harrisonii* Owen, 1861. J Vert Paleont 21 (suppl. 3)84A.

[35] Parish JC (2005) The Evolution and Palaeobiology of the Armoured Dinosaurs (Ornithischia: Ankylosauria). Unpublished PhD dissertation, University of Oxford, 471 pp.

[36] Galton PM (1974) Notes on *Thescelosaurus*, a conservative ornithopod dinosaur from the Upper Cretaceous of North America, with comments on ornithopod classification. J Paleont 48: 1048–1067.

[37] Hatcher JB, Marsh OC, Lull RS (1907). *The Ceratopsia*. Monographs of the United States Geological Survey 49

[38] Ostrom JH, McIntosh JS (1966) Marsh's dinosaurs: the collections from Como Bluff. New Haven: Yale University Press. 388 p.

[39] Lull RS, Wright NE (1942) Hadrosaurian dinosaurs of North America. Geol Soc Amer Spec Papers 40: 1–272.

[40] Maryanska T, Osmolska H (1968) Pachycephalosauria, a new suborder of ornithischian dinosaurs. Results of the Polish-Mongolian Palaeontological Expeditions. Palaeont. Pol. 19: 1–45.

[41] Hooley RW (1925) On the skeleton of *Iguanodon atherfieldensis* sp. nov. Quart J Geol Soc 81: 1–60.

[42] Lehman TM (1989) *Chasmosaurus mariscalensis*, sp. nov. a new ceratopsian dinosaur from Texas. J Vert Paleont 9: 137–162.

[43] Zhou SW (1984) The Middle Jurassic dinosaurian fauna from Dashanpu, Zigong, Sichuan. Vol 2: Stegosaurs. Sichuan: Sichuan Scientific and Technological Publishing House. 51 p.

[44] Coombs WP (1979) Osteology and myology of the hindlimb in the Ankylosauria (Reptilia Ornithischia). J Paleont 53: 666–684.

[45] Gilmore CW (1930) On dinosaurian reptiles from the Two Medicine Formation of Montana. US Natl Mus Proc 77 16: 1–39.

[46] Carpenter K (1990) Ankylosaur systematics: example using *Panoplosaurus* and *Edmontonia* (Ankylosauria: Nodosauridae). In: Carpenter K, Currie P, editors. Dinosaur Systematics: Perspectives and Approaches. New York: Cambridge University Press. pp 282–298.

[47] Lull RS (1921) The Cretaceous armored dinosaur, *Nodosaurus textilis* Marsh. Amer J Sci Ser 5 1: 97–125.

[48] Carpenter K, Kirkland JI, Burge D, Bird J (1999) Ankylosaurs (Dinosauria: Ornithischia) of the Cedar Mountain Formation, Utah, and their stratigraphic distribution. In Gillette D, editor. Vertebrate Paleontology in Utah. Utah Geol Sur, Misc Publ 99-1: 244–251.

[49] Ostrom JH (1970) Stratigraphy and paleontology of the Cloverly Formation (Lower Cretaceous) of the Bighorn Basin area, Wyoming and Montana. Peabody Mus Nat Hist Bul 35: 1–234.

[50] Norman DB (2010) A taxonomy of iguanodontians (Dinosauria: Ornithopoda) from the lower Wealden Group (Cretaceous: Valanginian) of southern England. Zootaxa 2489: 47–66.

[51] McDonald AT (2011) The taxonomy of species assigned to *Camptosaurus* (Dinosauria: Ornithopoda). Zootaxa 2783: 52–68.

[52] McDonald AT (2012) Phylogeny of basal iguanodonts (Dinosauria: Ornithischia): an update. PloS One 7 5: e36745 10.1371/journal.pone.0036745 PMC3358318 22629328

[53] Godefroit P, Codrea V, Weishampel DB (2009) Osteology of *Zalmoxes shqiperorum* (Dinosauria, Ornithopoda), based on new specimens from the Upper Cretaceous of Nălaţ-Vad (Romania). Geodiversitas 31 3: 525–553.

